# Supplementary material for: Selective CO2 reduction to CH3OH over atomic dual-metal sites embedded in a metal-organic framework with high-energy radiation
Source: Nat Commun. 2023 Aug 8;14:4767. doi: 10.1038/s41467-023-40418-3 (PMC10409780; doi:10.1038/s41467-023-40418-3)
Supplement: Supplementary file 1 — Supplementary Information [file 41467_2023_40418_MOESM1_ESM.pdf]

Supplementary Information

Selective CO_2 reduction to CH_3OH over atomic dual-metal sites embedded in a metal-organic framework with high-energy radiation

Changjiang Hu,[1] Zhiwen Jiang,[1] Qunyan Wu,[2] Shuiyan Cao,[3] Qiuhao Li,[1] Chong Chen,[1] Liyong Yuan,[2] Yunlong Wang,[1] Wenyun Yang,[4] Jinbo Yang,[4] Jing Peng,[5] Weiqun Shi,[2] Maolin Zhai,[5]* Mehran Mostafavi,[6]* Jun Ma[1,7]*

[1]Department of Materials Science and Technology, Nanjing University of Aeronautics and Astronautics Nanjing 211106, P. R. China.

[2]Laboratory of Nuclear Energy Chemistry, Institute of High Energy Physics, Chinese Academy of Sciences, Beijing 100049, P. R. China.

[3] Key Laboratory for Intelligent Nano Materials and Devices, College of Physics, Nanjing University of Aeronautics and Astronautics Nanjing 211106, P. R. China.

[4] State Key Laboratory for Mesoscopic Physics, School of Physics, Peking University, Beijing, 100871, PR China

[5]Radiochemistry and Radiation Chemistry Key Laboratory of Fundamental Science, College of Chemistry and Molecular Engineering, Peking University, Beijing 100871, P. R. China;

[6]Institut de Chimie Physique UMR8000, CNRS/Université Paris-Saclay, 91405 Orsay, France.

[7]School of Nuclear Science and Technology, University of Science and Technology of China, Hefei, Anhui 230026, P. R. China.

These authors contributed equally: Changjiang Hu, Zhiwen Jiang, and Qunyan Wu.

*.*Corresponding authors: junma@nuaa.edu.cn;*

mehran.mostafavi@universite-paris-saclay.fr;

mlzhai@pku.edu.cn;

Supplementary Table 1 Cu and Ni loadings of Cu SAs/UiO-66(Hf) and Ni SAs/UiO-66(Hf) and CuNi SAs/UiO-66(Hf).

Sample Name	Cu loading (wt%)	Ni loading (wt%)
Cu SAs/UiO-66(Hf)	1.8	0
Ni SAs/UiO-66(Hf)	0	0.42
CuNi SAs/UiO-66(Hf)	1.6	0.33

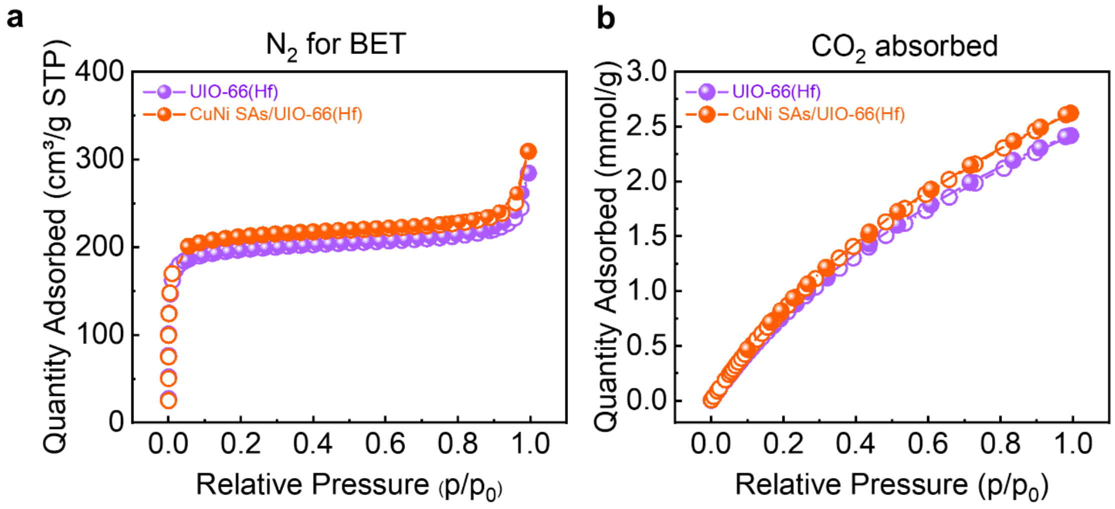

Supplementary Fig. 1 (a) N_2 and (b) CO_2 adsorption-desorption isotherms of CuNi SAs/UiO-66(Hf) and UiO-66(Hf).

Supplementary Table 2. S_{BET}, pore volume, and pore diameter of CuNi SAs/UiO-66(Hf) and UiO-66(Hf).

Sample	S_{BET} (m^2/g)	Pore Volume (cm^3/g)	Pore Diameter (nm)
UiO-66(Hf)	725.6	0.388	2.13
CuNi SAs/UiO-66(Hf)	756.1	0.478	2.5

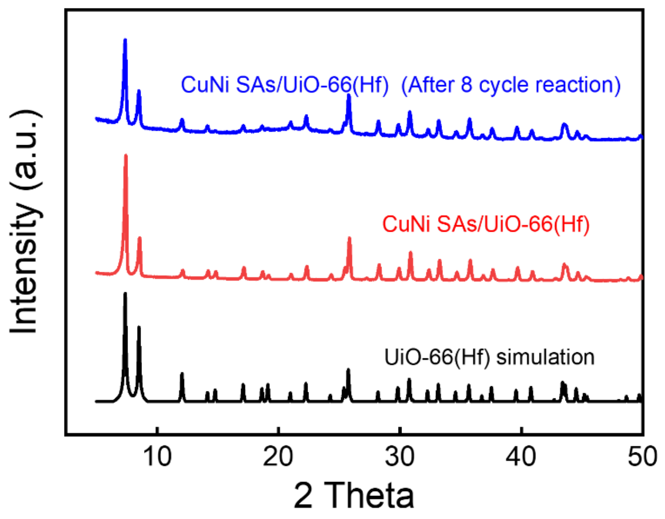

Supplementary Fig. 2 XRD patterns of CuNi SAs/UiO-66(Hf) before and after 40 kGy irradiation.

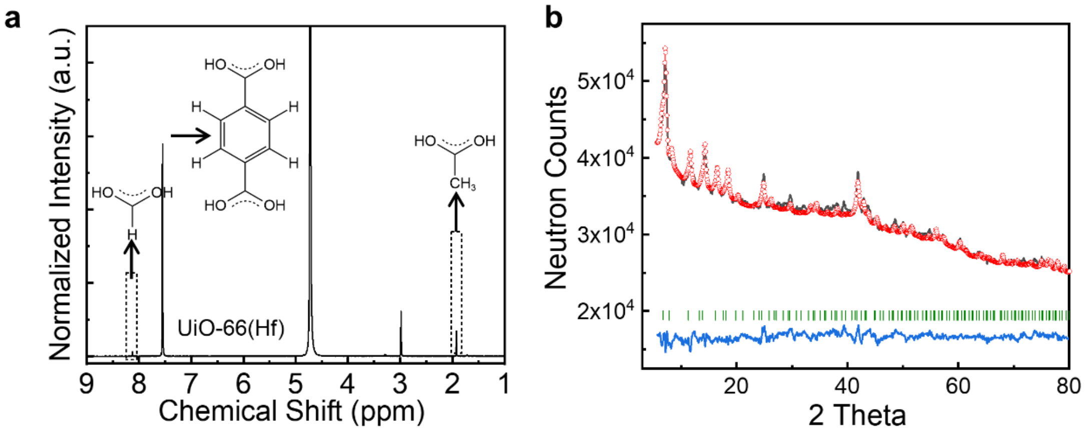

Supplementary Fig. 3 a, Dissolution ^1H NMR and **b,** Neutron powder diffraction spectra of UiO-66(Hf) and CuNi SAs/UiO-66(Hf).

Previous studies reported two types of defects in UiO-66: the absence of metal clusters and the absence of ligands. While many studies have focused on ligand deletion in monatomic systems, it is noteworthy that oxygen atoms linked to SBU (Secondary Building Units) serve as monatomic ligand sites[1,2]. Therefore, our study primarily investigated defective ligand deletion using liquid NMR spectroscopy as a starting point. Firstly, in the case of UiO-66(Hf) with ligand loss, oxygen atoms lacking ligands are likely supplied by two carboxylate ions ($HCOO^-$ and CH_3COO^-)[3,4], one of which originates from formate ions produced through DMF pyrolysis. Interestingly, our UiO-66 synthesis process did not involve the addition of acetate (CH_3COO^-) as a regulator, but the congruent presence of CH_3COO^- observed in our results aligns with those reported in the literature. During synthesis, acetate may come from impurities such as solvents, ligands, or metal salt, which may serve as a source of coordinating ions for ligand deletion in the MOF. The NMR data corroborated these pieces of assumptions.

Next, we refined the neutron diffraction data of UiO-66(Hf). It has been previously noted that X-ray data collection for UiO-66, which contains heavy metal atoms like Zr and Hf, exhibits high sensitivity towards these metal atoms but lacks sensitivity towards light atoms (C, H, O) in the absence of ligands[5]. We replaced the ligand with deuterated benzene-1,4-dicarboxylic acid to obtain better experimental data. Conversely, neutron diffraction provides improved sensitivity to the light atoms. Based on the collected powder neutron diffraction data, we initially assumed a fully saturated state for the atomic occupancy of elements in UiO-66(Hf) without any ligands. This means that the atomic occupancy was set to 100%, and the corresponding fitting parameters (goodness of fit data: R_p= 1.24, R_{wp}= 1.58, χ^2= 7.82) were obtained. However, as we defined the center of gravity of the defect as a ligand defect, the Hf periphery of the center, composed of $[Hf_6O_4(OH)_4]$, still exhibited 8 coordination oxygen atoms. We assumed that the missing oxygen atom, resulting from the absence of a partial dispenser, was provided by carboxylic acids ($HCOO^-$ and CH_3COO^-). Hence, the atomic occupancy

of Hf and O elements was assumed to be 100%. Our results revealed that the other ligand-contributed atoms (C H) occupied approximately 92% of UiO-66(Hf) (goodness of fit data: R_p= 1.23, R_{wp}= 1.57, χ^2= 7.71), resulting in better fitting results compared to those obtained without considering defects. These findings indicate the presence of ligand defects in UiO-66.

Supplementary Table 3. Structural parameters extracted from the Cu K-edge and Ni K-edge EXAFS fitting.

Sample	Scattering pair	CN	R(Å)	σ(10^{-3} Å^2)	ΔE$_0$(eV)	R factor
CuNi SAs/UiO-66(Hf)	Cu-O	2.64	1.93	4.5	4.16	0.009
CuNi SAs/UiO-66(Hf)	Ni-O	2.45	2.02	9.0	-4.12	0.007

S_0^2 is the amplitude reduction factor; CN is the coordination number; R is the interatomic distance (the bond length between central atoms and surrounding coordination atoms); σ is the Debye-Waller factor (a measure of thermal and static disorder in absorber-scatterer distances); ΔE$_0$ is edge-energy shift (the difference between the zero kinetic energy value of the sample and that of the theoretical model). The R factor is used to evaluate the goodness of the fitting.

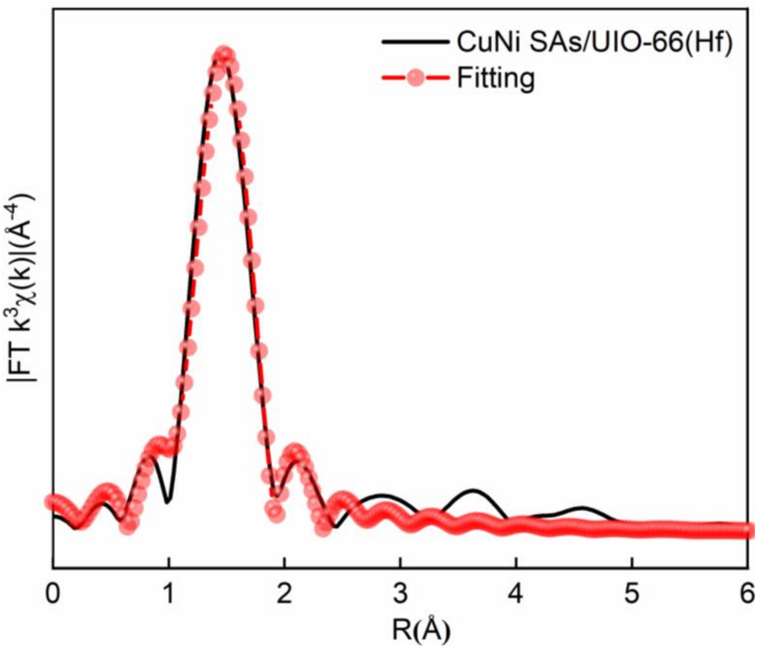

Supplementary Fig. 4 Cu K-edge EXAFS spectrum fitting of CuNi SAs/UiO-66(Hf).

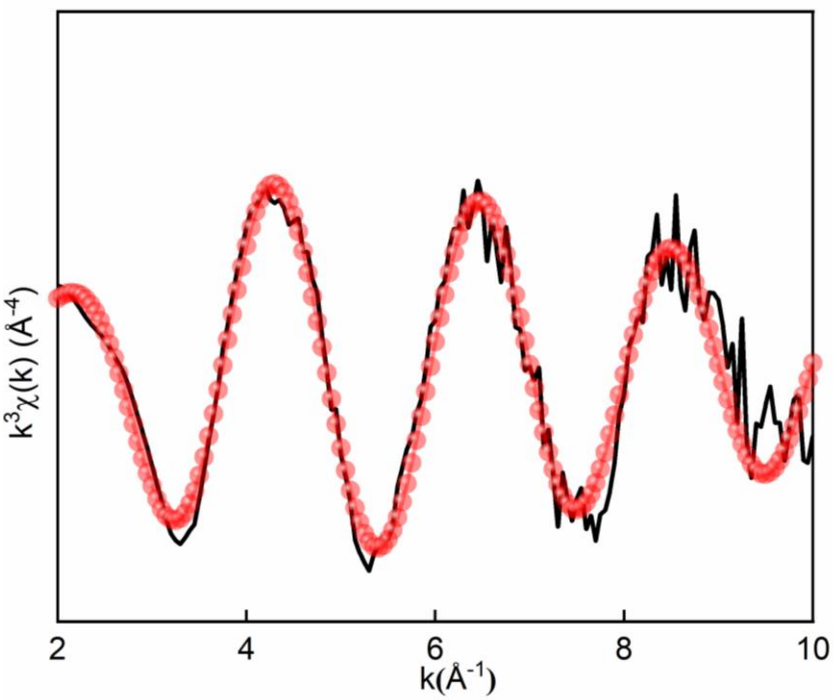

Supplementary Fig. 5 EXAFS fitting curves of the CuNi SAs/UiO-66(Hf) at Cu k space.

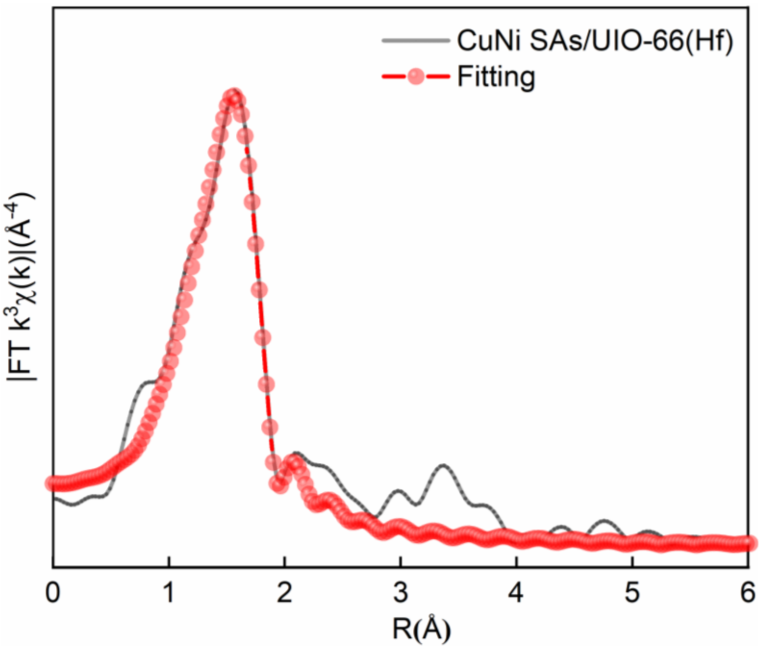

Supplementary Fig. 6 Ni K-edge EXAFS spectrum fitting of CuNi SAs/UiO-66(Hf).

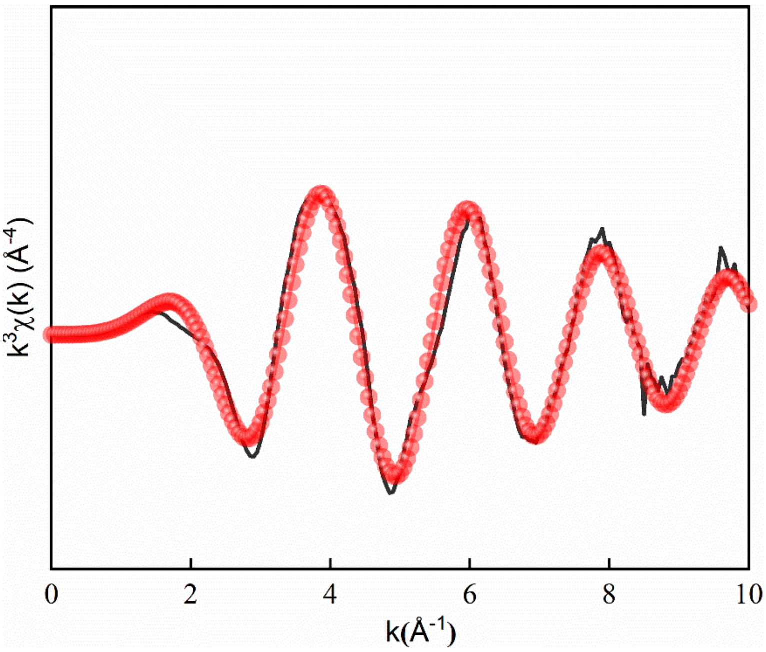

Supplementary Fig. 7 EXAFS fitting curves of the CuNi SAs/UiO-66(Hf) at Ni k space.

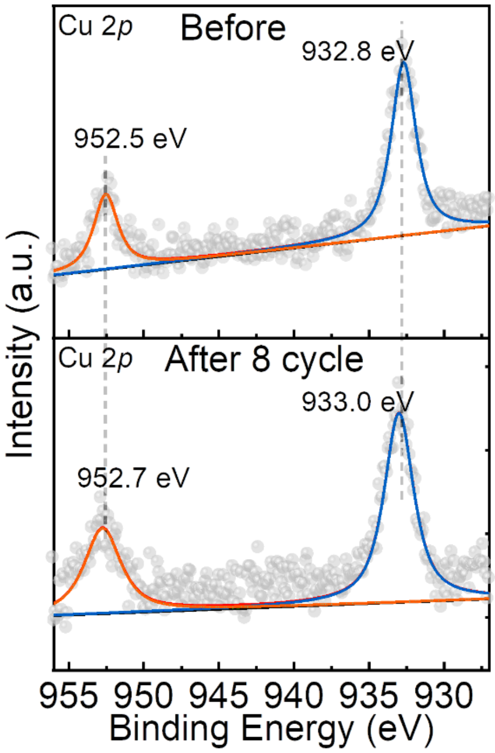

Supplementary Fig. 8 X-ray photoelectron spectroscopy spectra of Cu 2*p* in CuNi SAs/UiO-66(Hf) samples.

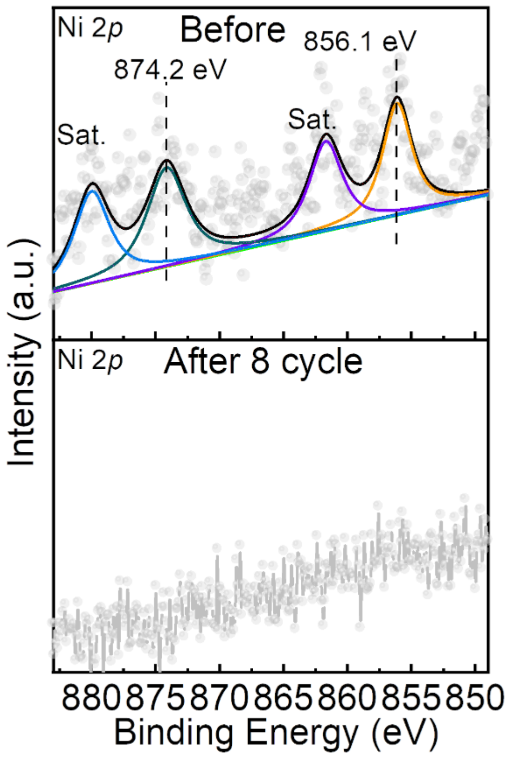

Supplementary Fig. 9 X-ray photoelectron spectroscopy spectra of Ni 2*p* in CuNi SAs/UiO-66(Hf) samples.

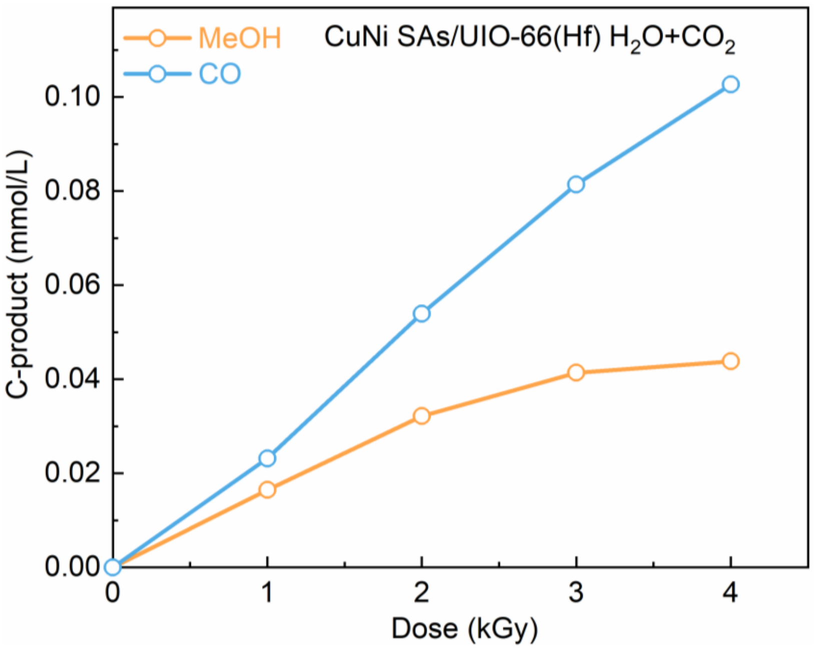

Supplementary Fig. 10 Yield of MeOH and CO with catalyst CuNi SAs/UiO-66(Hf) in CO_2 saturated solution under 1-4 kGy γ-ray irradiation.

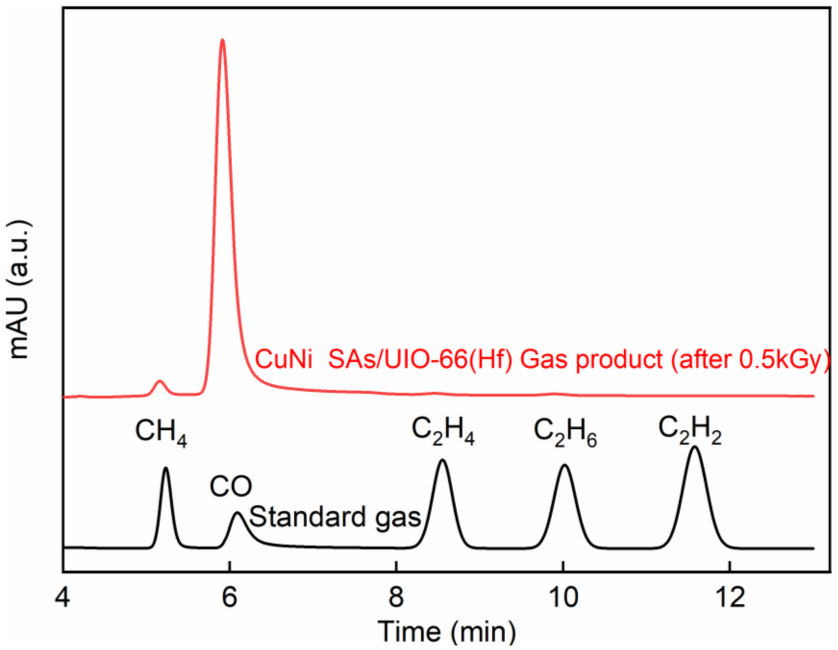

Supplementary Fig. 11 GC Spectra of CH_4, CO, C_2H_4, C_2H_6, and C_2H_2 with the standard gases of high purities (> 99.99%) composited of CO_2 (99%), H_2 (1%), CH_4 (101 ppm), CO (58 ppm), C_2H_4 (100 ppm), C_2H_6 (100 ppm), C_2H_2 (100 ppm), and catalytic reaction gas sample after 0.5 kGy irradiation.

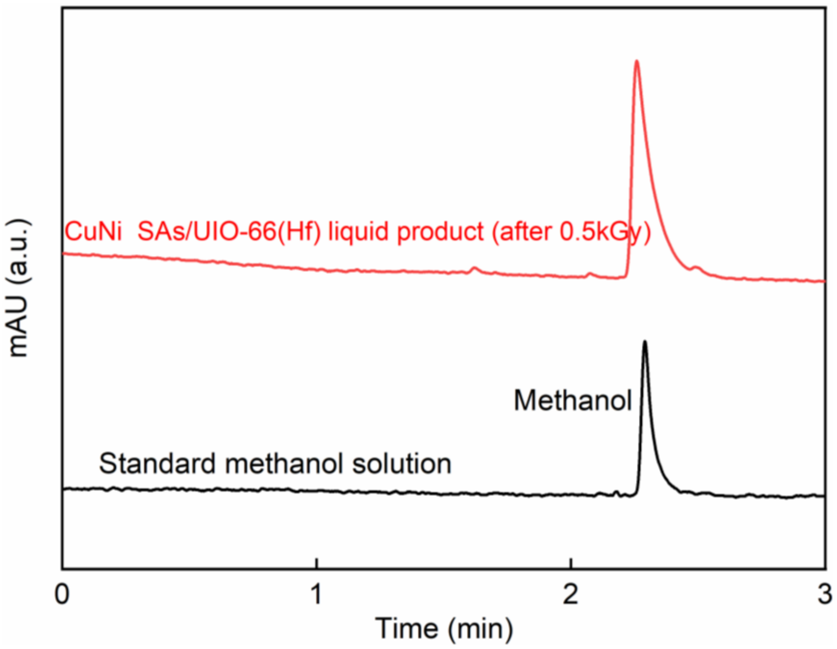

Supplementary Fig. 12 GC Spectra of CH_3OH with methanol solution (0.1 mM) and the catalytic reaction gas sample after 0.5 kGy irradiation.

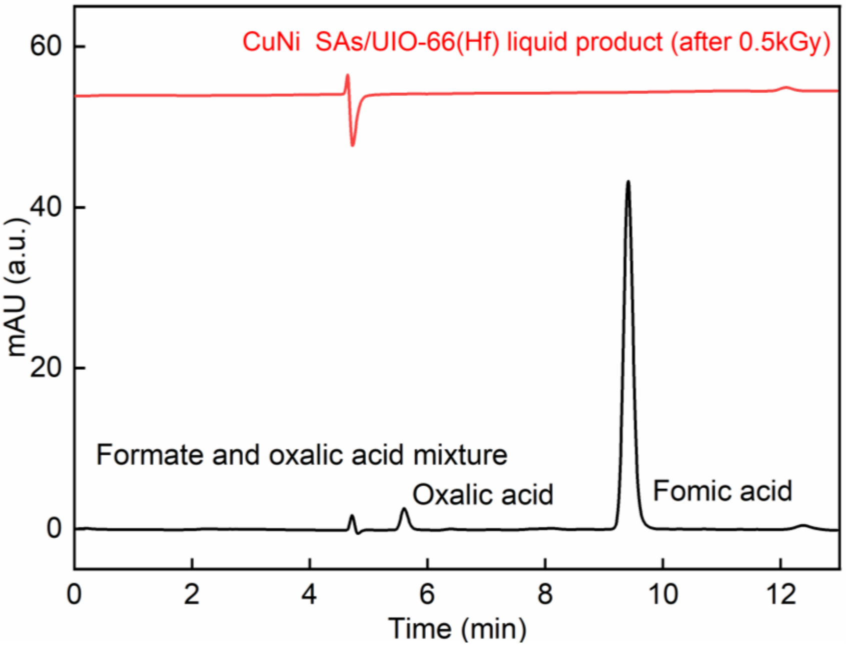

Supplementary Fig. 13 HPLC Spectra of CH_3OH with formic acid and oxalic acid, as well as catalytic reaction gas sample after 0.5 kGy irradiation.

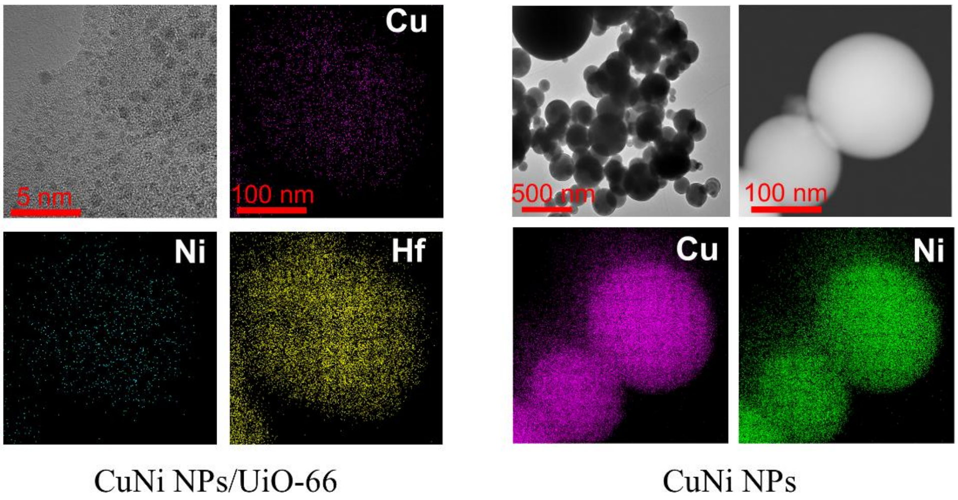

Supplementary Fig. 14 HRTEM and EDS-mapping of CuNi NPs/UiO-66(Hf) and nanoparticles (NPs) CuNi.

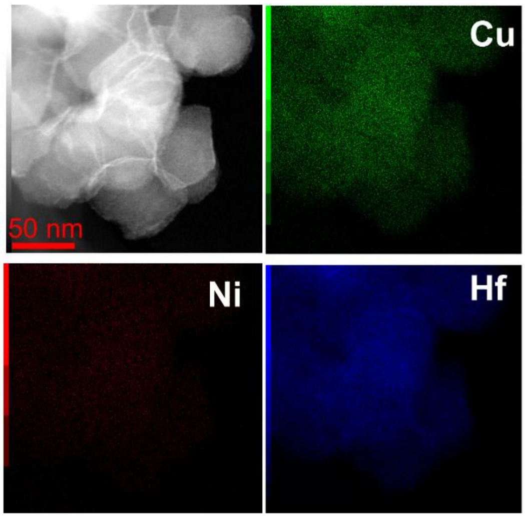

Supplementary Fig.15 TEM EDS mapping and XPS of CuNi SAs/UiO-66(Hf) after 8 times stability test.

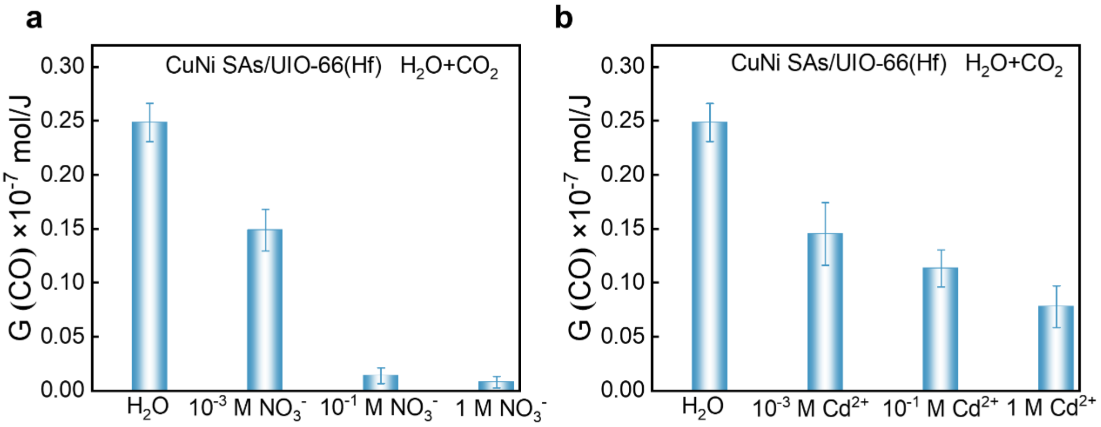

Supplementary Fig. 16 The decreasing *G*-value of CO with increasing concentration of **a** NO_3^- and **b** Cd^{2+} ions.

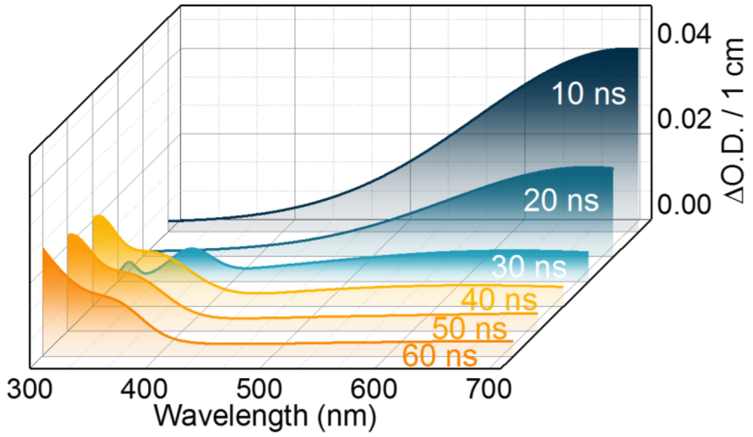

Supplementary Fig. 17 3D stereograph of transient absorption spectra recorded after electric pulse at different times with CO_2-saturated 0.1 M formate aqueous solution[6].

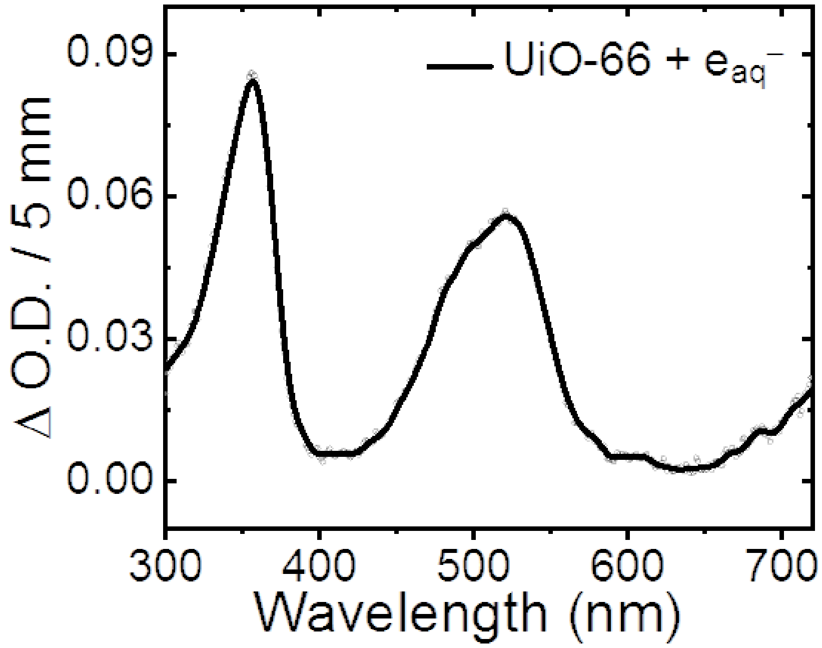

Supplementary Fig. 18 The transient absorption spectrum at 600 ns in 0.1 M tert-butanol solution containing 0.25 mg/mL UiO-66.

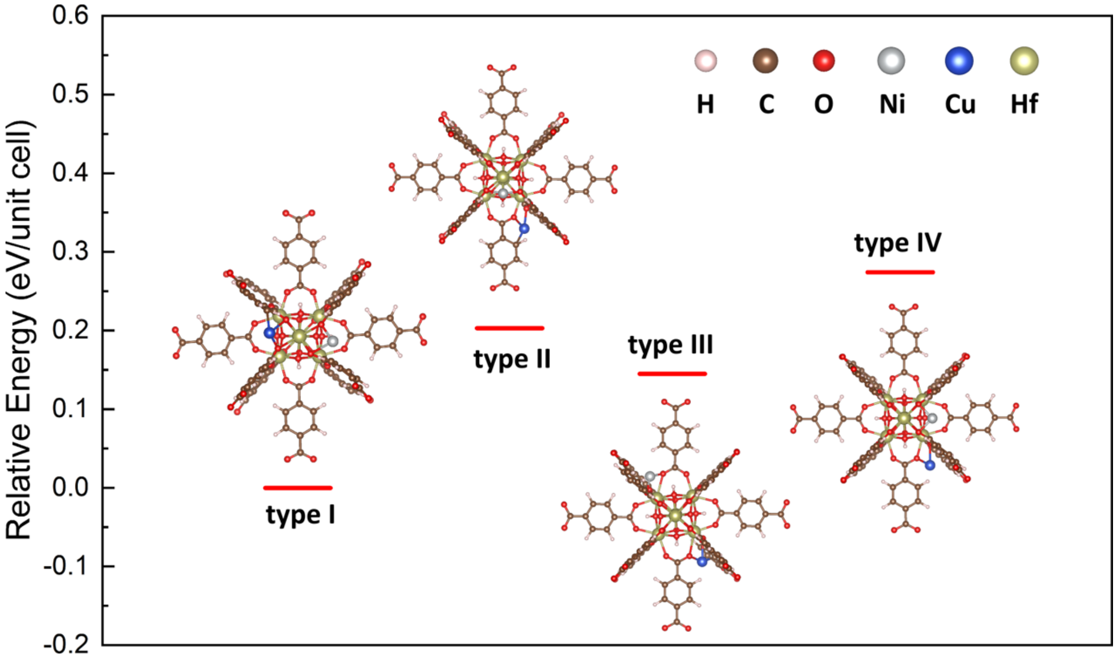

Supplementary Fig. 19 Views showing the four types of Cu and Ni single atom combined with UiO-66(Hf), Hf oxide cluster of UiO-66(Hf), and Cu single atom link.

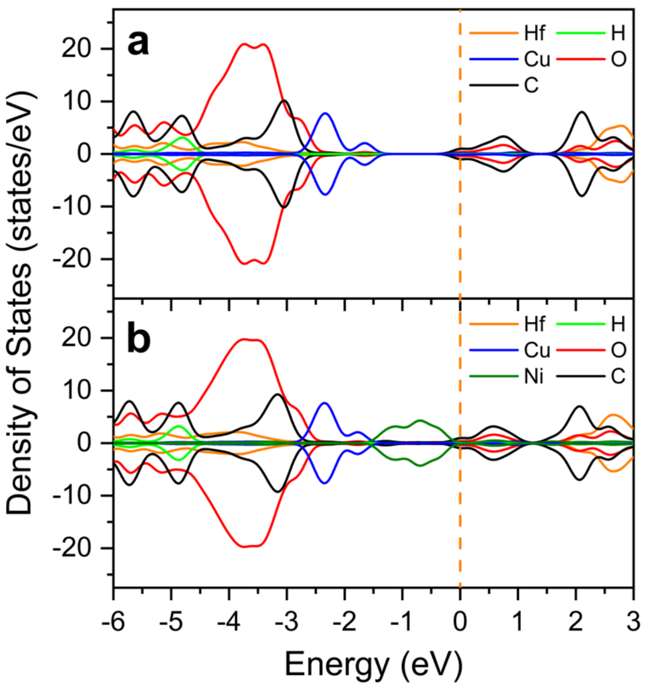

Supplementary Fig. 20 PDOS plots for **a.** Cu SAs/UIO-66(Hf) and **b.** CuNi SAs/UiO-66(Hf).

Supplementary References:

1 Ma, X. *et al.* Modulating coordination environment of single-atom catalysts and their proximity to photosensitive units for boosting MOF photocatalysis. *J. Am. Chem. Soc.* **143**, 12220-12229 (2021).

2 Abdel-Mageed, A. M. *et al.* Highly active and stable single-atom Cu catalysts supported by a metal–organic framework. *J. Am. Chem. Soc.* **141** 5201-5210 (2019).

3 Tan, K. *et al.* Defect termination in the UiO-66 family of metal-organic frameworks: the role of water and modulator. *J. Am. Chem. Soc.* **143**, 6328-6332 (2021).

4 Shearer, G. C. *et al.* Defect engineering: tuning the porosity and composition of the metal-organic framework UiO-66 via modulated synthesis. *Chem. Mater.* **28**, 3749-3761 (2016).

5 Wu, H. *et al.* Unusual and highly tunable missing-linker defects in zirconium metal–organic framework UiO-66 and their important effects on gas adsorption. *J. Am. Chem. Soc.* **135**, 10525-10532 (2013).

6 Buxton, G. V. & Sellers, R. M. Acid dissociation constant of the carboxyl radical. Pulse radiolysis studies of aqueous solutions of formic acid and sodium formate. *J. Chem. Soc., Faraday Trans. 1* **69**, 555-559, (1973).
